# Primary Burkitt Lymphoma of the Fourth Ventricle in an Immunocompetent Young Patient

**DOI:** 10.1155/2014/630954

**Published:** 2014-08-31

**Authors:** Abdulrahman Alabdulsalam, Syed Z. A. Zaidi, Imran Tailor, Yasser Orz, Sadeq Al-Dandan

**Affiliations:** ^1^Department of Pathology, King Abdulaziz Medical City, Riyadh 14611, Saudi Arabia; ^2^Department of Adult Hematology/BMT, CCC, King Fahad Medical City, Riyadh 11525, Saudi Arabia; ^3^Department of Adult Neurosurgery, NNI, King Fahad Medical City, Riyadh 11525, Saudi Arabia; ^4^Department of Pathology, King Fahad Medical City, Riyadh 11525, Saudi Arabia

## Abstract

Primary Burkitt lymphoma of the central nervous system (CNS) is rare, with only few cases reported in the literature. An 18 year-old immunocompetent male presented with multiple cranial nerves palsies and was found to have a mass predominantly in the 4th ventricle of the brain. Tumor was surgically removed and showed morphological and immunohistochemical features consistent with Burkitt lymphoma. The patient responded very well to anthracycline based chemotherapy with high dose methotrexate (HD MTX) and intrathecal (IT) chemotherapy delivered by Ommaya reservoir. Primary Burkitt lymphoma of the CNS is a rare entity that poses differential diagnostic challenge with other small round blue cell tumors.

## 1. Introduction

Burkitt lymphoma is high-grade B-cell lymphoma of germinal center origin, and it has rarely been reported as a primary brain neoplasm [1]. We present a case of a primary Burkitt lymphoma of the brain predominantly involving the 4th ventricle in an immunocompetent adult. The patient was primarily treated by surgical debulking (as it was initially suspected to be medulloblastoma) followed by chemotherapy and intraventricular chemotherapy through Ommaya reservoir resulting in complete resolution of the tumor.

## 2. Case Presentation

**Figure 1:**
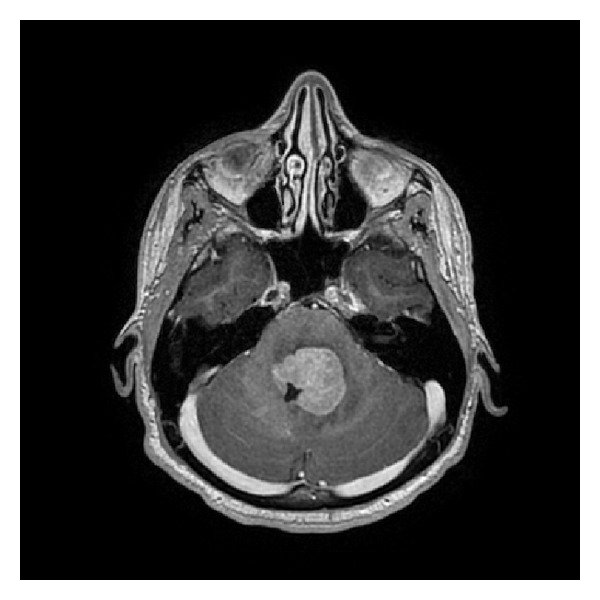
T1-weighted, gadolinium-enhanced axial magnetic resonance image, showing a well-demarcated intensely enhancing subependymal lesion occluding the lumen of the fourth ventricle and causing moderate peritumoral edema.

An 18-year-old male, not known to have any medical illness, was admitted to Aseer Central Hospital, Abha, Saudi Arabia, with a history of progressive ataxia for 4 weeks. He also had double vision, facial asymmetry, tinnitus, and dysphagia during the last 2 weeks prior to presentation. On examination, the patient was alert, conscious, and oriented, and his vital signs were stable. He was found to have bilateral palsies of the fourth cranial nerve, as well as palsies of the left 7th, 9th, and 10th cranial nerves, in addition to mild cerebellar ataxia. His CT scan of the brain revealed a posterior fossa mass. The patient was transferred to King Fahad Medical City, Riyadh, for further investigation and management. He had performance status of 2 on Eastern Cooperative Oncology Group (ECOG) scale. A magnetic resonance imaging of the brain (Figure 1) was done and showed a homogeneously enhancing, well-demarcated subependymal tumor outlining the fourth ventricle, occluding its lumen, and extending down into the foramen of Magendie. It measured 3 × 3 × 3.5 cm and it had mass effect, but no true invasion of the brainstem was noted. Moderate peritumoral edema was seen within the neighboring brainstem and the left cerebellum. Similar, however, smaller lesions were noted; the first one was in the floor of the third ventricle, anterior to the aqueduct, which measured 12 × 6 mm, and the other was seen in spinal intradural extramedullary location filling in the lateral recess of the C4 level on the right side. Blood tests including CBC differential count, U&E, LFT, and uric acid were normal. Lactate dehydrogenase (LDH) was also normal (206 U/L). Serological tests results for hepatitis B, hepatitis C, and HIV were unremarkable. Quantitative immunoglobulins were also normal. Lymphocyte subset analysis revealed normal absolute CD4 positive T-helper cells (1.73 × 10^9^/L). Blood film review did not show any abnormal cells. The cerebrospinal fluid (CSF) cells count showed RBC 526/cumm and WBC 1/cumm. CSF cytology examination did not reveal any abnormal cell. CSF protein was not high (0.1 G/L).

**Figure 2:**
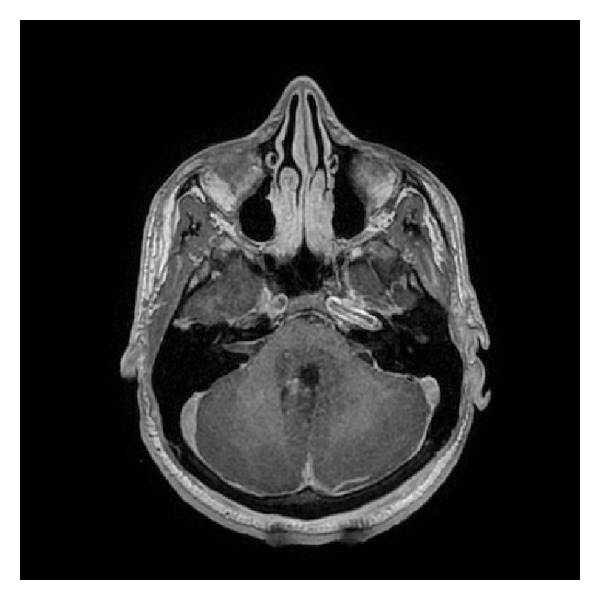
T1-weighted, immediate postoperative, gadolinium-enhanced axial magnetic resonance image, showing gross total resection with small enhancing changes along the posterior aspect of the surgical resection cavity.

**Figure 3:**
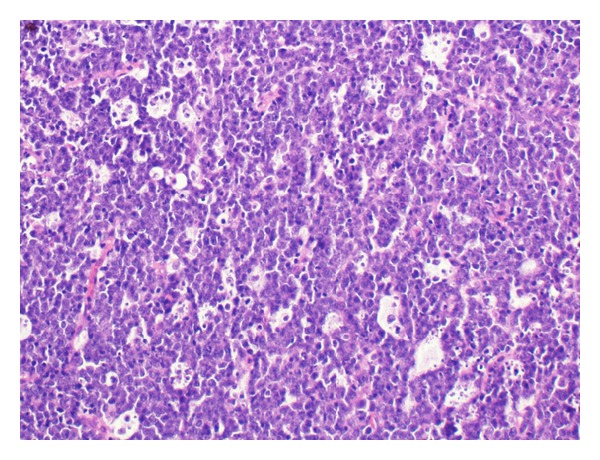
Tangible body macrophages dispersed among sheets of discohesive cells, giving the typical “starry sky” morphologic appearance (H & E stain, ×200 magnification).

**Figure 4:**
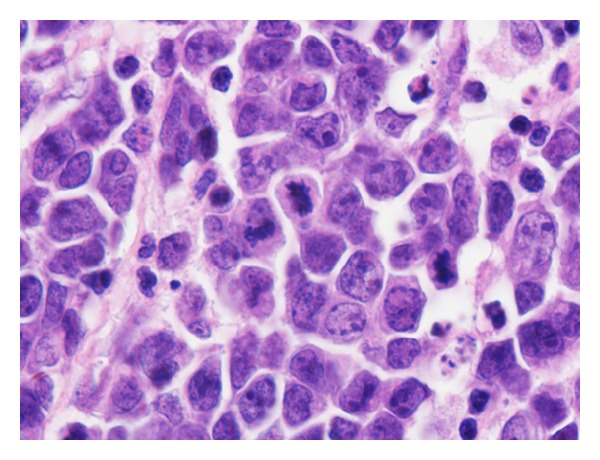
The lymphoma cells are squared off. Mitotic figures and apoptotic bodies are evident (H & E stain, ×1000 magnification with oil).

**Figure 5:**
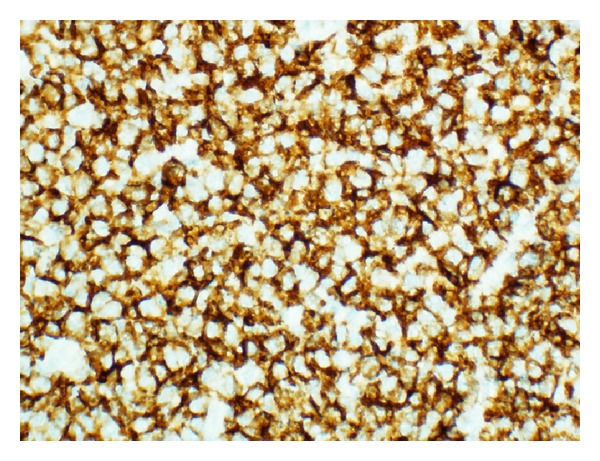
Strong and diffuse immunoreactivity for CD 20 (CD 20 Immunostain, ×400 magnification).

**Figure 6:**
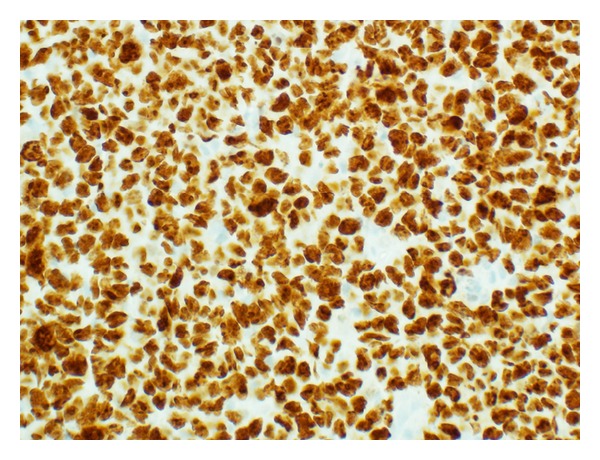
Ki-67 proliferation index of >99% (MIB-1 immunostain, ×400 magnification).

The patient underwent suboccipital craniotomy and transvermian approach for tumor removal where a gross total resection of the intraventricular lesion was achieved with a curative intent except lesions in third ventricle and cervical spinal cord (Figure 2). The frozen section showed a round blue cell tumor, initially favoring medulloblastoma. On microscopic examination of the permanent and additional sections, the tumor showed sheets of medium-sized discohesive cells displaying high nucleus to cytoplasm ratio and brisk mitotic activity. Lots of apoptotic bodies as well as tangible body macrophages were seen, giving rise to “starry sky” appearance (Figure 3). Some cells demonstrated squared-off borders, reminiscent of molding (Figure 4); however, no neuropil or salt and pepper nuclear chromatin pattern were seen. There were no eccentric nuclei or eosinophilic cytoplasmic globules to suggest atypical teratoid/rhabdoid tumor (ATRT). Immunohistochemical staining showed the tumor cells to be positive for CD45, CD20, CD79, CD10, and Bcl-6 (Figure 5). Tumor cells were negative for Bcl-2, CD3, CD30, TdT, and Cyclin D1. The Ki67 labeling index was more than 99% (Figure 6). In situ hybridization for Epstein-Barr virus-encoded RNA (EBER) was negative. The negative reaction to synaptophysin and GFAP argued against medulloblastoma and glioma, respectively. The positive INI and negative Cam5.2 and SMA ruled out ATRT. Cytogenetic study confirmed t(8:14) by FISH technique performed on a paraffin embedded tissue section as all of the scored nuclei revealed IGH/CMYC rearrangement signals. CT scans of neck, chest, and abdomen; whole body PET scan MRI studies; and bone marrow biopsy (including FISH analysis for c-myc gene rearrangements) did not show any sign of extracranial lymphoma; hence, our final diagnosis was primary Burkitt lymphoma of the CNS.

The patient was then treated with chemotherapy and is being followed up by multidisciplinary teams. He was offered R-CHOP with HD MTX (3 gram/m^2^) chemotherapy regimen every 21 days (HD MTX typically being offered on day 10 of 21-day cycle) and triple intrathecal chemotherapy injections (TITs) containing methotrexate, cytarabine, and hydrocortisone through Ommaya reservoir. The patient completed 6 cycles of R-CHOP+HD MTX and 9 injections of TITs. In view of his poor performance status and lack of enough data on appropriate treatment regimen in the literature, the team decided to choose this regimen. It was planned to offer whole brain radiotherapy (WBRT) at the end as consolidation. Two months after the chemotherapy, an MRI of brain showed a good response to the therapy with near complete resolution of the postsurgical residual tumor in the posterior fossa and brainstem, complete resolution of the posterior third ventricle focus, and a near complete resolution of the C3-C4 intradural extramedullary focus. At the end of treatment he developed leukoencephalopathy changes on follow-up MRI albeit he had remarkable improvement in his tumor with no evidence of further recurrence. Consequently, the planned WBRT was not given and the chemotherapy treatment was hence stopped and he underwent extensive rehabilitative physiotherapy.

On the last followup of the patient (18 months after the surgery), he was able to walk independently with a cane but still had multiple neurological deficits of the cranial nerves, including residual facial palsy, altered taste sensation, residual dysarthria, and a mild gait ataxia, although he is independent with daily activities. He also had multiple Botox injection in bilateral spastic medial rectus muscle to reduce the double vision in addition to eyelid surgeries for bilateral Bell's palsy. However, no evidence of recurrence of the lymphoma has been noted.

## 3. Discussion

We report a case of primary Burkitt lymphoma of the brain in an immunocompetent adult patient of 18 years of age. The main bulk of the tumor was in the fourth ventricle. Burkitt lymphoma is known to involve the CNS as part of systemic disease; however, to our knowledge; there are only 20 reported cases as primary Burkitt lymphoma of the central nervous system (Table 1) [1–19]. Only five cases of Burkitt lymphoma presented as posterior fossa masses [4, 6, 7, 15, 18].

Central nervous system (CNS) involvement of non-Hodgkin lymphoma (NHL) occurs as a primary or secondary disease. Primary central nervous system lymphoma (PCNSL) accounts for 3% of all newly diagnosed brain tumors and 2-3% of all cases of NHL. Surveillance, epidemiology, and end results (SEER) database indicates that the incidence of this tumor may be rising among patients 65 years of age and older [20]. Diffuse large B-cell lymphoma accounts for 90% of PCNSL, followed by T-cell lymphomas and mucosa associated lymphoid tissue (MALT) lymphoma [8]. The etiology of PCNSL is not fully understood and the lymphomagenesis is largely undefined. Significant risk factors for PCNSL include acquired or congenital immunodeficiency states. PCNSL is also an AIDS-defining condition associated with a very low CD4 T-cell count (<50 cells/mL) [21]. The association between AIDS-related PCNSL and Epstein-Barr virus (EBV) expression in the tumor is near 100%. By contrast, EBV is rarely detected in PCNSL of immunocompetent patients, suggesting a different pathogenesis in each group [22].

Among immunocompetent patients, PCNSL has a median age at diagnosis of 56 years and a male-to-female ratio of 1.2–1.7 : 1. In newly diagnosed PCNSL, lesions are solitary in 65% and multifocal in 35% [22]. Our patient was relatively younger, immunocompetent, had a large mass in posterior fossa in addition to smaller foci, and did not harbor EBV in the tumor. All of these features argue in favor of sporadic form of Burkitt lymphoma.

Prognosis of PCNSL is poor with rapid progression if left untreated. The international extranodal lymphoma study group (IELSG) described 5 poor prognostic parameters in PCNSL: (1) age older than 60 years; (2) ECOG performance status >1; (3) elevated LDH; (4) high CSF protein concentration; and (5) tumor location within the deep regions of the brain (periventricular, basal ganglia, brainstem, and/or cerebellum). The 2-year overall survival rates for patients with 0 to 1, 2 to 3, or 4 to 5 of these adverse risk factors are 80%, 48%, or 15%, respectively [23]. Our patient had 2 risk factors (performance status of 2 and tumor location) implying a probability of 48% for two-year overall survival.

All therapeutic modalities except high-dose methotrexate (HD-MTX) are subject to controversy, especially whole brain radiotherapy (WBRT) and intrathecal chemotherapy. There is a consistent recommendation among review articles and international guidelines, including those of the US National Comprehensive Cancer Network, is that planned resections of PCNSL should be discouraged [24]. This is based on the evidence that aggressive surgery may increase the risk of postoperative neurologic deficit and provides no impact on survival compared with biopsy alone [25, 26].

Recent investigators are challenging this paradigm. According to their study, when controlled for the number of lesions, aggressive resection of PCNSL correlated with better progression-free survival with the regimen studied [24, 27]. A recent retrospective study of the German PCNSL Study Group-1 (GPSG-1) trial, a large randomized phase III study comprising of 526 patients with PCNSL, the progression-free survival (PFS) and overall survival (OS) were significantly shorter in biopsied patients compared with patients with subtotal or gross total resections. This difference in outcome was not due to age or Karnofsky performance status (KPS) [24]. Accordingly, in individualized cases (well-circumscribed tumors with significant mass effect, in which tumor debulking is deemed feasible with low risk of neurologic deficit) aggressive surgical resections may provide significant clinical benefit including immediate relief of mass effect, facilitating the rapid tapering of glucocorticoids, and intuitively eliminating the cell populations with drug resistance potential [22].

Interestingly, unpublished observations of our clinical colleagues (Syed Z. A. Zaidi and Imran Tailor) at King Fahad Medical City, Riyadh, also suggest that Burkitt Lymphoma patients who reached our institution after (so-called erroneous) surgical debulking of tumor in other organs, for example, hemicolectomy for huge masses involving colon, have showed excellent outcome after relatively less intensive chemotherapy using modified Vanderbilt chemotherapy + rituximab protocol.

Unfortunately, the highest level of evidence for chemotherapy treatment of PCNL comes from phase 2 clinical trials. The likely reason is the rarity of this illness and poor performance status in the majority of patients. As mentioned earlier, the majority tend to be diffuse large B-cell lymphoma; very little is known about natural history, treatment, and prognosis of primary CNS Burkitt lymphoma [8]. Systemic Burkitt lymphoma differs from other aggressive lymphomas as it is treated by intense chemotherapy regimens akin to acute lymphoblastic leukemias such as Hyper-CVAD regimen or CODOX-M-IVAC unlike other high-grade lymphomas [28]. In the literature, the patients with primary CNS Burkitt lymphoma are treated with HD MTX based regimen [15, 29]. This distinct biology and poor performance status of patient made us choose RCHOP with HD MTX and further 9 cycles of triple IT chemotherapy through Ommaya reservoir. IT chemotherapy is generally not recommended when HD MTX is used; however, we did give intraventricular chemotherapy as this patient had ventricular lesions [30]. When HD MTX with high-dose cytarabine (HD ARA C) was used along with whole brain radiotherapy in an international multicentric phase 2 randomized trial comprising of 79 patients with PCNL (including one case of Burkitt lymphoma), it was found that patients who received HD MTX and HD ARA C with WBRT had better response than those who received HD MTX and WBRT, although toxicity was increased in former. In HD MTX/HD ARA C/WBRT arm complete remission rates and overall response rates were 46% and 69%, respectively, compared to 18% and 40% in HD MTX/WBRT arm [29].

Our reported case of primary CNS Burkitt lymphoma in an immunocompetent adult posed unique diagnostic and therapeutic challenges including choice for optimal chemotherapy regimen and role of surgical debulking. This case has many other peculiar rarities including very young age and involvement of posterior fossa and fourth ventricle. The patient is alive and independent with daily activities and is in complete remission for the last 18 months, although he has some residual neurological deficits including facial weakness, dysarthria, and mild ataxia.

This case report should stimulate thoughts for larger studies to explore the impact of debulking surgeries in lymphoma patients, especially in primary CNS lymphomas, and also define optimal chemotherapy regimens not only in PCNSL but also in primary CNS Burkitt lymphoma.

## Figures and Tables

**Table 1:** Reported cases of primary Burkitt lymphoma in central nervous system.

Case number	Author	Year	Age	Gender	Location	Previous status/immunity
1	Valsamis et al. [1]	1976	3 m	Male	Both temporal tips and partial temporal area	Positive serology for EBV

2	Gigormini et al. [2]	1981	11 y	Male	Temporooccipital area	Excised astrocytoma, 6 months before presentation with Burkitt lymphoma

3	Kobayashi et al. [3]	1984	55 y	Female	Right temporoparietal area	Not available

4	Hegedus [4]	1984	50 y	Female	Brainstem and cerebellum	Nothing significant/competent

5	Tekkok et al. [5]	1991	5 y	Male	Frontobasal parasellar area	Nothing significant/competent

6	Toren et al. [6]	1994	6 y	Female	? Midbrain	Nothing significant/competent

7	Späth-Schwalbe et al. [7]	1999	40 y	Male	Cerebellum and pons	Nothing significant/competent

8	Monabati et al. [8]	2002	49 y	Female	Right parietal lobe	Iron deficiency anemia/competent

9	Shehu [9]	2003	8 y	Male	Left temporal area and right lateral orbit	Not available

10	Gobbato et al. [10]	2006	38 y	Male	Right frontotemporoparietal subdural area	AIDS

11	Abel et al. [11]	2006	50 y	Male	Central thalamus and right thalamus	Not available

12	Kozáková et al. [12]	2008	60 y	Female	Sellar (pituitary)	Nothing significant/competent

13	Takasu et al. [13]	2010	71 y	Male	Hypothalamic third ventricle	Known case of inactive TB/competent

14	Gu et al. [14]	2010	75 y	Female	Third and left lateral ventricles	Resolved cerebral infarction/competent

15	Lim et al. [15]	2011	43 y	Female	Medulla oblongata	Not available

16	Jiang et al. [16]	2011	14 y	Male	Right lateral ventricle	Nothing significant/competent

17	Akhaddar et al. [17]	2012	13 y	Female	Right infratemporal area	Nothing significant/competent

18	Yoon et al. [18]	2012	10 y	Male	Suprasellar, cerebellum, 3rd ventricle	Not available

19	Yoon et al. [18]	2012	32 m	Male	Sellar area, extend to orbit/sphenoid	Not available

20	Jiang et al. [19]	2012	69 y	Male	Right temporal and occipital lobes, cervical spine, and cauda equina	Nothing significant/competent
